# Concomitant full-thickness cartilage lesions do not affect patient-reported outcomes at minimum 10-year follow-up after ACL reconstruction

**DOI:** 10.1007/s00167-021-06757-8

**Published:** 2021-10-09

**Authors:** Katherine Wang, Cathrine N. Eftang, Svend Ulstein, Asbjørn Årøen, Rune B. Jakobsen

**Affiliations:** 1 Department of Orthopedic Surgery, Akershus University Hospital, Oslo, Norway; 2 Faculty of Medicine, University of Oslo, Boks 1072 Blindern, 0316 Oslo, Norway; 3 Oslo Sports Trauma Research Center, Norwegian School of Sports Sciences, Oslo, Norway; 4 Department of Pathology, Akershus University Hospital, Oslo, Norway; 5 Department of Health Management and Health Economics, Institute of Health and Society, University of Oslo, Oslo, Norway; 6 Faculty of Medicine, Institute of Clinical Medicine, University of Oslo, Oslo, Norway

**Keywords:** Anterior cruciate ligament, Reconstruction, Cartilage lesion, Outcome, KOOS

## Abstract

**Purpose:**

To compare patients with a concomitant full-thickness cartilage lesion and anterior cruciate ligament (ACL) injury to patients with an isolated ACL injury at 10–15 years post ACL reconstruction.

**Methods:**

This is a longitudinal follow-up of a cohort of 89 patients that were identified in the Norwegian National Knee Ligament Registry and included in the index study in 2007. The study group consisted of 30 patients that underwent ACL reconstruction and had a concomitant, isolated full-thickness cartilage lesion (International Cartilage Repair Society [ICRS] grade 3–4). Each study patient was matched with two control patients who underwent ACL reconstruction but had no cartilage lesions (ICRS grade 1–4) (*n* = 59). At a median follow-up of 10.2 years (range 9.9–15.6), 65 patients (74%) completed the Knee Injury and Osteoarthritis Outcome Score (KOOS), which was the main outcome measure, resulting in 23 pairs after matching.

**Results:**

At a follow-up of 10–15 years after ACL reconstruction, no significant differences in KOOS were found between patients with a concomitant full-thickness cartilage lesion and patients without cartilage lesions. There was also no significant difference between the two groups when comparing the change over time in KOOS scores from preoperative to follow-up. Both groups showed significant improvement in all KOOS subscales from preoperative to follow-up, except for in the Symptoms subscale for the control group. The greatest improvement was in the QoL subscale for the study group.

**Conclusion:**

ACL-reconstructed patients with a full-thickness cartilage lesion did not report worse outcomes at 10–15 years after surgery compared with patients with an isolated ACL injury. Our findings support that there is no long-term negative effect of a concomitant cartilage lesion in an ACL-reconstructed knee. These findings should be considered when discussing treatment and informing about the expected long-term outcome after ACL reconstruction to patients with such combined injuries.

**Level of evidence:**

II.

## Introduction

Anterior cruciate ligament (ACL) reconstruction is one of the most commonly performed orthopedic procedures and a well-established treatment option with multiple reports on the long-term outcomes, both subjective and objective [5, 16, 22, 27]. ACL injuries are often associated with other injuries in the knee and the choice of treatment for these injuries is not always clear. Cartilage lesions can be found in 16–46% of knees undergoing primary ACL reconstructions [7]. There is yet to be a consensus for whether, and how, these lesions should be treated and rehabilitated [12, 15, 20, 26, 30, 36, 37, 39, 47].

The effect of a concomitant cartilage lesion on the long-term outcomes for patients who have undergone an ACL reconstruction is indecisive [10, 13]. In studies focusing on patient-reported outcomes, a cartilage lesion at the time of the index ACL reconstruction has been shown to be a risk factor for significantly poorer subjective outcomes at follow-up times of 10 years or more [2, 17, 23, 24, 34, 35]. However, although the changes in standardized subjective knee scores were statistically significant, they were not always clinically relevant. Similar results have been found in studies looking at radiographic osteoarthritis (OA) in ACL-reconstructed knees. Cartilage lesions are a significant risk factor for developing radiographic OA, and also increase the risk of symptomatic radiographic OA [2, 8, 23–25, 27].

This study is the fourth report on a longitudinal follow-up of a cohort of ACL-reconstructed patients with concomitant cartilage lesions of International Cartilage Repair Society (ICRS) grade 3 or 4 and a matched control group without cartilage lesion. The cohort was described in an index study by Hjermundrud et al. where they found no effect of the cartilage lesion on preoperative KOOS scores [19]. At a median of 2.1 years post reconstruction, Røtterud et al. reported that patients with concomitant cartilage lesions had significantly worse outcomes [29]. However, at a median of 6.3 years of follow-up, Ulstein et al. found no negative effect of concomitant cartilage lesions compared with matched controls [40].

The hypothesis of this study was that at a minimum 10-year follow-up, patients with ACL reconstruction and a concomitant cartilage lesion would not have significantly worse outcomes compared with a matched control group. With this study, the aim was to increase knowledge of the long-term prognosis after ACL reconstruction in patients with a concomitant cartilage lesion and thereby improve information on the expected prognosis to patients. To our knowledge, this is one of very few long-term prognostic studies in this area.

## Materials and methods

The National Knee Ligament Registry (NKLR) in Norway prospectively monitors the outcome of knee ligament surgeries [14] with the Knee Injury and Osteoarthritis Outcome Score (KOOS) filled in by the patient preoperatively, and at 2-, 5-, and 10-year follow-ups.

### Knee injury and osteoarthritis outcome score

The KOOS is a self-administered questionnaire for patients and is considered valid, reliable, and responsive to ACL and cartilage lesions [4, 9, 11]. It consists of five subscales: pain, symptoms, activities of daily living (ADL), function in sport and recreation (sport/rec), and knee-related quality of life (QoL). Each subscale ranges from zero (worst) to 100 (best). The KOOS QoL subscale is considered to be the most sensitive for ACL-injured patients and was defined as the primary outcome [28]. A difference of 8–10 points in a subscale is considered to be the minimal perceptible clinical improvement [28].

### Patient inclusion

A search performed in the NKLR identified 4849 primary ACL reconstructions in 2004–2007. Of these, 30 patients met the following inclusion criteria: a full-thickness cartilage lesion (ICRS grade 3 or 4), age less than 40 years, less than 12 months between ACL injury and reconstruction, no associated ligament or meniscus injury, no previous knee surgery, and a complete preoperative KOOS questionnaire. Each of these 30 patients in the study group was matched with two control patients with an isolated ACL injury and no cartilage lesion of any ICRS grade. The control patients had to meet the same inclusion criteria as the study group and were matched for age, gender, time from injury to reconstruction, and type of graft. The strict inclusion criteria and matching were intended to isolate the cartilage lesion as the only factor distinguishing the two groups and thereby minimize influence of other possible factors on knee function and degenerative development.

The full-thickness cartilage lesions of the study group were distributed as follows: 20 (67%) in the medial tibiofemoral compartment, 6 (20%) in the lateral tibiofemoral compartment, and 4 (13%) in the patellofemoral compartment. Twenty-two (73%) had a lesion measuring 2 cm^2^ or less, and eight (27%) were greater than 2 cm^2^. Only seven (23%) of the patients had a cartilage procedure performed at the time of the reconstruction: four patients had a debridement procedure, and three patients had a micro-fracture procedure. The cartilage lesions of the remaining patients were left untreated.

### Follow-up

**Fig. 1:**
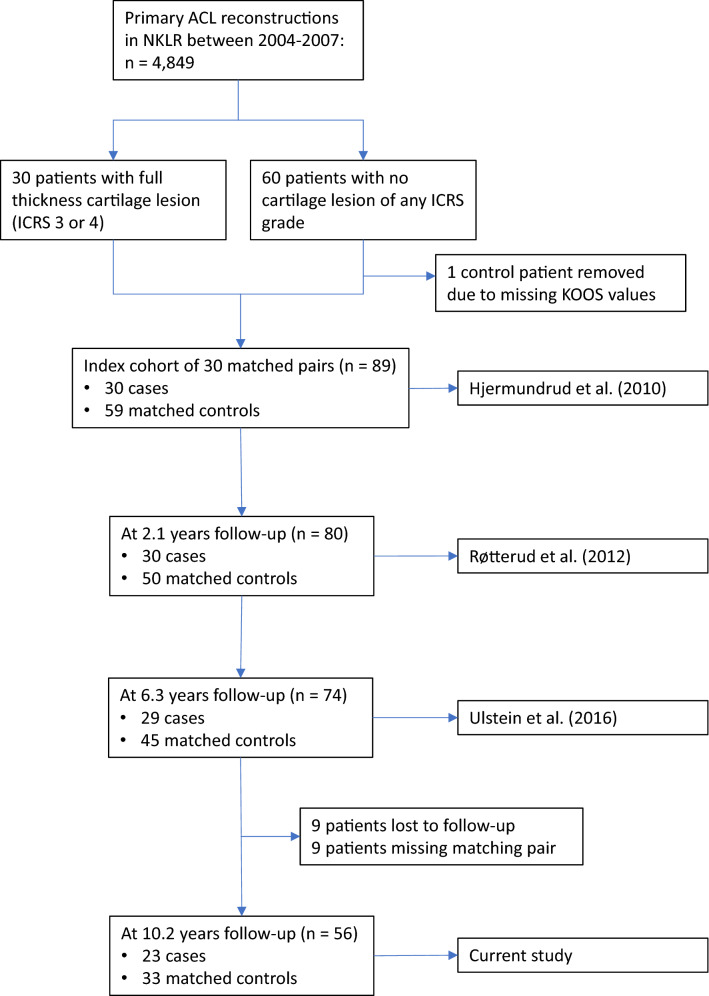
Flow-chart illustrating patient inclusion and participation from index study by Hjermundrud et al. through subsequent follow-ups up to current study [19, 29, 40]. Anterior cruciate ligament (ACL); National Knee Ligament Registry (NKLR); International Cartilage Repair Society (ICRS); Knee injury and Osteoarthritis Outcome Score (KOOS)

At a median of 10.2 years (range 9.9–15.6, *n* = 52), KOOS data were collected from NKLR. Patients lacking 10-year follow-up data in the NKLR were sent the KOOS questionnaire and Tegner activity scale up to three times by postal mail. They were further contacted by telephone to fill in the questionnaire verbally if there was still no response by post. Sixty-five KOOS forms were collected with a response rate of 74%. After matching cases with controls, 23 matched pairs remained (*n* = 23 cases, *n* = 33 controls) (Fig. 1). The patients that were excluded at previous follow-ups or lost to this follow-up were removed from the preoperative KOOS data. In addition to KOOS questionnaires, Tegner activity score (*n* = 31), height and weight (*n* = 56), and smoking status (*n* = 53) were also collected at follow-up. All patients were cross-referenced in the Norwegian Arthroplasty Registry to determine if any had undergone a total knee replacement.

### Statistical analysis

Comparisons between the study and control group were performed using paired sample *t* tests, and all mean differences and mean changes measured by KOOS were given with a 95% confidence interval (CI). If KOOS was available for both of the matched controls, the data were regarded as clustered and the average score of the two patients was used in the analysis. Level of significance was defined as *p* ≤ 0.05. The power analysis identified 26 pairs as necessary at follow-up to detect a change in KOOS QoL subscale of 10 points given a power of 0.80, a significance level of 0.05, and a standard deviation (SD) of the difference between the study group and the control group of 17.2, which was the SD of the difference between the groups preoperatively [19, 29, 40]. To determine if the groups were comparable, a Student’s *t* test for body mass index (BMI) and a chi-square test for smoking status were performed as these variables were not matched for in the initial pairings.

IBM SPSS (Statistical Package of Social Sciences) software version 25.0 was used for all statistical analyses.

## Results

The study group and the control group were comparable regarding age, time from injury to operation, gender distribution, graft type, smoking status, and Tegner score at follow-up (Table 1). There was no significant difference between the groups regarding BMI or smoking status. Non-responders were mostly male (19 male, 4 female), but did not differ significantly in any other characteristics from the responders with regards to age at injury, time from injury to operation or in any of the preoperative KOOS subscale scores.

**Table 1:** Characteristics of the study groups at follow-up

	Study group (*n* = 23)	Control group (*n* = 33)
Age (years)^a^ (*n* = 52)	38.1 (7.9)	38.2 (8.7)
Follow-up (years)^a^ (*n* = 52)	11.4 (2.1)	11.1 (1.8)
Time from injury to surgery (months)^a^ (*n* = 56)	5.6 (2.5)	5.1 (2.4)
Gender^c^ (*n* = 56)		
Females	7 (30)	13 (39)
Males	16 (70)	20 (61)
Right/left (*n* = 56)	12/11	19/14
Body mass index^a^ (*n* = 56)	25.9 (3.0)	25.9 (4.3)
Graft type^c^ (*n* = 56)		
Hamstring tendons	14 (61)	20 (61)
Patella tendon/other	9 (39)	13 (39)
Smoking status^c^ (*n* = 53)		
Non-smokers	18 (82)	26 (84)
Tegner activity level^b^ (*n* = 31)	4 (1–7)	4 (1–6)

^a^Mean and (standard deviation)

^b^Median and (range)

^c^Number and (percentages)

**Fig. 2:**
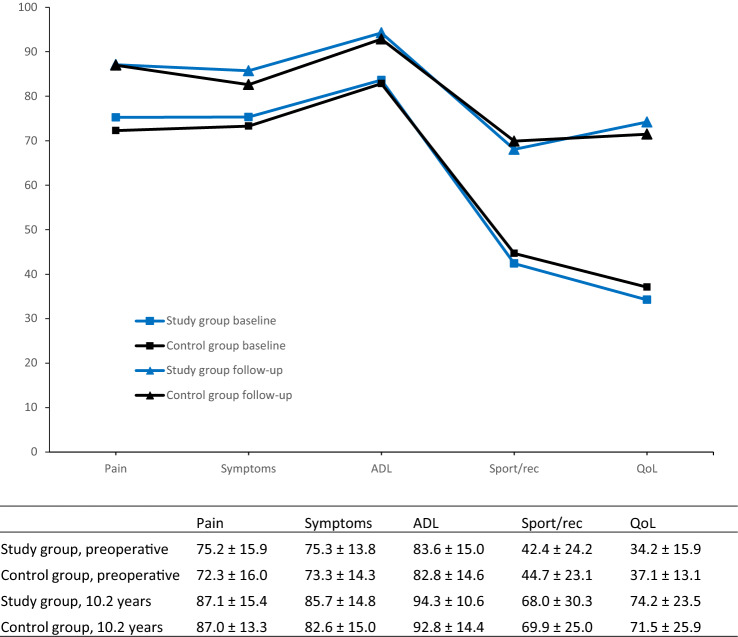
Mean Knee Injury and Osteoarthritis Outcome Score (KOOS) of the study group (ACL injury with full-thickness cartilage lesion) and the control group (isolated ACL injury) at preoperative and a median of 10.2 years of follow-up after ACL reconstruction with standard deviation. Activities of daily living (ADL); Sports and recreation (Sport/rec); Quality of life (QoL); Anterior cruciate ligament (ACL)

The mean scores preoperatively and at follow-up for the study group (*n* = 23) (ACL injury with concomitant full-thickness cartilage lesion) and the control group (*n* = 33) (isolated ACL injury) are shown for each KOOS subscale in Fig. 2. After removal of the non-responders from the preoperative data, there were no significant differences between the study and control group preoperatively, nor at the 10-year follow-up (Table 2). There were also no significant differences between the two groups when comparing the change over time in KOOS scores from preoperative to follow-up. From 6.3 to 10.2 years, the patients with cartilage lesion and ACL reconstruction continued to improve with the largest improvement in the QoL subscale (31.8 ± 10.5 at 6.3 years to 40.0 ± 24.3 at 10.2 years) (Table 3). The mean improvement was also clinically relevant for all subscales, except the symptoms’ subscale for the control group. None of the patients were identified in the Norwegian Arthroplasty Registry as having undergone a knee replacement procedure. In a sensitivity analysis, we removed the six pairs not available for the long-term follow-up from the five-year dataset. This did not affect the results of the longitudinal analysis.

**Table 2:** Mean difference in Knee Injury and Osteoarthritis Outcome Score (KOOS) between the study group and the control group preoperatively and at follow-up of 10.2 years with change over time

KOOS subscales	Preoperative		Follow-up		Change over time	
	Mean difference (95% CI)	*p*-value	Mean difference (95% CI)	*p*-value	Mean difference (95% CI)	*p*-value
Pain	3.0 (− 7.2 to 13.2)	(n.s.)	0.1 (− 9.6 to 9.8)	(n.s.)	− 2.8 (− 11.1 to 5.4)	(n.s.)
Symptoms	2.0 (− 6.5 to 10.6)	(n.s.)	3.1 (− 6.1 to 12.3)	(n.s.)	1.1 (− 9.5 to 11.7)	(n.s.)
ADL	0.8 (− 7.2 to 8.8)	(n.s.)	1.4 (− 6.9 to 9.7)	(n.s.)	0.6 (− 9.3 to 10.5)	(n.s.)
Sport/rec	− 2.3 (− 17.6 to 13.0)	(n.s.)	− 1.8 (− 20.4 to 16.7)	(n.s.)	0.4 (− 15.4 to 16.3)	(n.s.)
QoL	− 2.9 (− 11.1 to 5.4)	(n.s.)	2.7 (− 12.8 to 18.3)	(n.s.)	5.6 (− 13.9 to 25.0)	(n.s.)

*n* = 23 case–control pairs at all points

Mean difference = study group minus control group

Change over time = follow-up minus preoperative

Confidence interval (CI); level of significance (p-value); not significant (n.s.); activities in daily living (ADL); quality of life (QoL)

**Table 3:** Mean change over time between preoperative scores and mean follow-up of 10.2 years in Knee Injury and Osteoarthritis Outcome Score (KOOS) of the study group and the control group

KOOS subscales	Study group (*n* = 23)		Control group (*n* = 33)	
	Mean change over time (95% CI)	*p*-value	Mean change over time (95% CI)	*p*-value
Pain	11.8 (6.0–17.7)	< 0.001	14.7 (6.9–22.4)	0.001
Symptoms	10.4 (2.7–18.1)	0.010	9.3 (− 0.1 to 18.7)	(n.s.)
ADL	10.6 (5.0–16.2)	0.001	10.0 (1.0–19.0)	0.031
Sport/rec	25.7 (15.1–36.3)	< 0.001	25.2 (13.0–37.5)	0.001
QoL	39.9 (29.4–50.5)	< 0.001	34.3 (20.2–48.6)	< 0.001

**Fig. 3:**
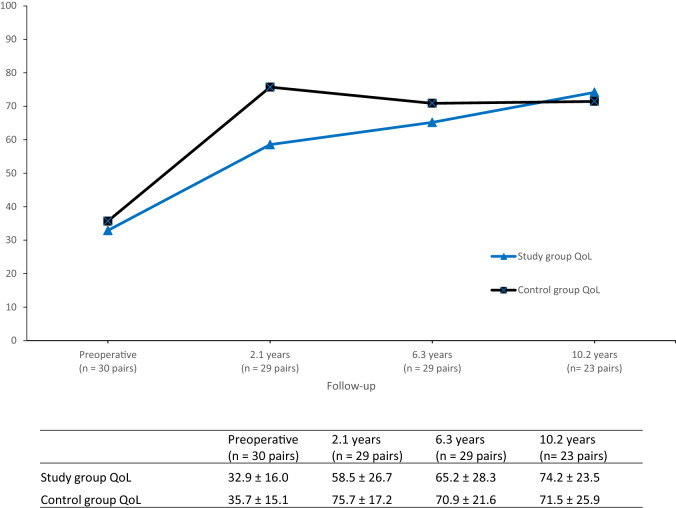
Knee Injury and Osteoarthritis Outcome Score (KOOS) quality of life (QoL) subscale for the study group (ACL injury with full-thickness cartilage lesion) and the control group (isolated ACL injury) at preoperative and all follow-ups after ACL reconstruction with standard deviation. Preoperative data from Hjermundrud et al. [19], 2.1 years from Røtterud et al. [29], and 6.3 years from Ulstein et al. [40]

Figure 3 shows the change in KOOS scores over time with the pre-operative baseline as the starting point. The QoL outcomes show a more constant improvement for the study group, while the control group improves rapidly in the short term with some deterioration in the mid- to long term.

## Discussion

The main finding of the present study is that concomitant full-thickness cartilage lesions identified at the time of ACL reconstruction do not significantly affect patient-reported outcomes more than 10 years after surgery.

This finding further supports the results of the previous report on this cohort, where Ulstein et al. found no negative effect of the concomitant full-thickness cartilage lesion on patient-reported outcomes at a median of 6.3 years of follow-up [40]. The patients with a cartilage lesion and ACL reconstruction continued to improve in all KOOS subscales, with the largest improvement in the QoL subscale (31.8 ± 10.5 to 40.0 ± 24). The control group had improved scores in the pain, ADL and QoL subscales, but no improvement in the Symptoms and Sport/rec subscales. The control group also showed a minimal increase of 1.3 points in the QoL subscale. The characteristics of the patients did not change with regards to BMI and Tegner activity level from 6.3 years to the current follow-up, which only allows for speculation to the causality behind the convergence of scores between groups at 6.3 years and in the present study from the divergence reported at 2.1 years of follow-up. The 6.3 years of follow-up did surprisingly find significantly more OA in the control group, which may have explained the slight reduction in scores in this group over time. We did not assess radiographic OA in this study, but differences in OA is unlikely to explain the continued improvement in the long term in the study group.

There are few studies focusing specifically on the long-term (≥ 10 years) prognostic effects of a cartilage lesion in ACL-injured patients. Balasingam et al., Hanypsiak et al. and Widuchowski et al. found no statistically significant differences in patient-reported outcomes when comparing patients with cartilage lesion and ACL injury to patients with isolated ACL injury at 10 years, 12 years, and 15 years, respectively [1, 18, 46]. In studies analyzing multiple parameters to identify risk factors for inferior long-term outcomes, there is largely a consensus that a cartilage lesion present at the time of primary ACL reconstruction represents a significant risk factor for poorer outcomes. Oiestad et al., Lebel et al., Murray et al., Spindler et al., Cantin et al., and Senorski et al. all found a negative influence of cartilage lesions on OA progression, QoL and International Knee Documentation Committee (IKDC) score [8, 17, 21, 23, 24, 35]. Similarly, Shelbourne et al. found statistically significantly lower subjective IKDC scores if the patient with cartilage injury also had less than normal motion [34].

Furthermore, the results demonstrate that knee patients with the combination of cartilage injury and ACL injury improve regardless of the treatment of the cartilage injury similarly to patients without concomitant cartilage injury. However, in several trials of surgical treatment of cartilage injuries, patients with combined injuries have been included at the time of ACL reconstruction [3, 6, 31–33, 41–44]. This raises the question whether the improvement seen in these trials may in part be due to the ACL reconstruction and not the cartilage treatment. One may question if such patients should be included in clinical trials on surgical cartilage repair techniques as they likely have a different prognosis than knees with a cartilage injury alone.

The main strengths of this study are the narrow inclusion criteria and strict matching of control patients. By matching in pairs for potential confounders, the exposed (cartilage lesion) group and the unexposed (isolated ACL injury) group will be less likely to have differences in the distribution of known confounders. However, those restrictions might limit the generalizability of the results. Although data for 65 patients (74% response rate) were collected, only 56 of these were included in the statistical analyses due to the matching of patients in pairs. This resulted in 23 pairs, which was less than the 26 pairs determined by the original power analysis, increasing the risk of a type-II error. Furthermore, there was a considerable expansion in standard deviations over time indicating the need for a larger sample size. The small sample size also did not allow for study patients who received treatment for their cartilage lesion to be separated into a third group for comparison, and the question of whether concomitant cartilage lesions should be treated at the time of ACL reconstruction continues to be a complicated topic. Further limitations of this study include that subsequent knee surgeries in the period from the previous to the current follow-up were not registered, and X-rays for OA could not be examined as the data collection collided with the restrictions due to the COVID-19 pandemic. However, none of the patients, including non-responders, were identified as having a total knee arthroplasty in the Norwegian Arthroplasty Register. Our cohort also included a majority of smaller lesions, however, a larger study from the NKLR has shown the prognosis of small (< 2 cm^2^) and large lesions (> 2 cm^2^) combined with ACL injury to be similar, thus supporting the generalizability of this long-term study [38].

The cumulative findings from this cohort show that a concomitant full-thickness cartilage lesion present at the time of ACL reconstruction can initially negatively affect patient-reported outcomes in the short term, but also that the effect appears to decrease in the long term. Interestingly, the study group with cartilage lesions continued to improve in KOOS scores in the mid- to long term despite most of the cartilage lesions remaining surgically untreated. This is valuable information that should be discussed with the patient in the preoperative stage, as it has been shown that evidence of cartilage damage was independently associated with worse patient and surgeon expectations regarding outcome after an ACL reconstruction [45]. These findings should be considered when informing patients with such combined injuries with regards to whether the cartilage lesion should be treated surgically and give realistic expectations regarding the expected outcome after ACL reconstruction.

## Conclusion

ACL-reconstructed patients with a full-thickness cartilage lesion did not report inferior outcomes at 10–15 years after surgery compared with patients with an isolated ACL injury and no cartilage injury. The longitudinal follow-up on this cohort suggests that a cartilage lesion will negatively affect patient-reported outcomes in the short term, but the effect will diminish in the long term.

## References

[1] Balasingam S, Sernert N, Magnusson H, Kartus J (2018). Patients with concomitant intra-articular lesions at index surgery deteriorate in their knee injury and osteoarthritis outcome score in the long term more than patients with isolated anterior cruciate ligament rupture: a study from the swedish national anterior cruciate ligament register. Arthroscopy.

[2] Barenius B, Ponzer S, Shalabi A, Bujak R, Norlén L, Eriksson K (2014). Increased risk of osteoarthritis after anterior cruciate ligament reconstruction: a 14-year follow-up study of a randomized controlled trial. Am J Sports Med.

[3] Basad E, Ishaque B, Bachmann G, Stürz H, Steinmeyer J (2010). Matrix-induced autologous chondrocyte implantation versus microfracture in the treatment of cartilage defects of the knee: a 2-year randomised study. Knee Surg Sports Traumatol Arthrosc.

[4] Bekkers JE, de Windt TS, Raijmakers NJ, Dhert WJ, Saris DB (2009). Validation of the knee injury and osteoarthritis outcome score (KOOS) for the treatment of focal cartilage lesions. Osteoarthr Cartil.

[5] Bodkin SG, Werner BC, Slater LV, Hart JM (2020). Post-traumatic osteoarthritis diagnosed within 5 years following ACL reconstruction. Knee Surg Sports Traumatol Arthrosc.

[6] Brittberg M, Recker D, Ilgenfritz J, Saris DBF (2018). Matrix-applied characterized autologous cultured chondrocytes versus microfracture: five-year follow-up of a prospective randomized trial. Am J Sports Med.

[7] Brophy RH, Zeltser D, Wright RW, Flanigan D (2010). Anterior cruciate ligament reconstruction and concomitant articular cartilage injury: incidence and treatment. Arthroscopy.

[8] Cantin O, Lustig S, Rongieras F, Saragaglia D, Lefèvre N, Graveleau N (2016). Outcome of cartilage at 12years of follow-up after anterior cruciate ligament reconstruction. Orthop Traumatol Surg Res.

[9] Collins NJ, Misra D, Felson DT, Crossley KM, Roos EM (2011). Measures of knee function: international knee documentation committee (IKDC) subjective knee evaluation form, knee injury and osteoarthritis outcome score (KOOS), knee injury and osteoarthritis outcome score physical function short form (KOOS-PS), knee outcome survey activities of daily living scale (KOS-ADL), lysholm knee scoring scale, oxford knee score (OKS), western ontario and mcmaster universities osteoarthritis index (WOMAC), activity rating scale (ARS), and tegner activity score (TAS). Arthritis Care Res (Hoboken).

[10] Diermeier T, Rothrauff BB, Engebretsen L, Lynch AD, Ayeni OR, Paterno MV (2020). Treatment after anterior cruciate ligament injury: panther symposium ACL Treatment consensus group. Knee Surg Sports Traumatol Arthrosc.

[11] Engelhart L, Nelson L, Lewis S, Mordin M, Demuro-Mercon C, Uddin S (2012). Validation of the knee injury and osteoarthritis outcome score subscales for patients with articular cartilage lesions of the knee. Am J Sports Med.

[12] Everhart JS, DiBartola AC, Swank K, Pettit R, Hughes L, Lewis C (2020). Cartilage damage at the time of anterior cruciate ligament reconstruction is associated with weaker quadriceps function and lower risk of future ACL injury. Knee Surg Sports Traumatol Arthrosc.

[13] Filardo G, de Caro F, Andriolo L, Kon E, Zaffagnini S, Marcacci M (2017). Do cartilage lesions affect the clinical outcome of anterior cruciate ligament reconstruction? A systematic review. Knee Surg Sports Traumatol Arthrosc.

[14] Granan LP, Bahr R, Steindal K, Furnes O, Engebretsen L (2008). Development of a national cruciate ligament surgery registry: the Norwegian national knee ligament registry. Am J Sports Med.

[15] Gudas R, Gudaite A, Mickevicius T, Masiulis N, Simonaityte R, Cekanauskas E (2013). Comparison of osteochondral autologous transplantation, microfracture, or debridement techniques in articular cartilage lesions associated with anterior cruciate ligament injury: a prospective study with a 3-year follow-up. Arthroscopy.

[16] Hamrin Senorski E, Svantesson E, Baldari A, Ayeni OR, Engebretsen L, Franceschi F (2019). Factors that affect patient reported outcome after anterior cruciate ligament reconstruction-a systematic review of the Scandinavian knee ligament registers. Br J Sports Med.

[17] Hamrin Senorski E, Svantesson E, Spindler KP, Alentorn-Geli E, Sundemo D, Westin O (2018). Ten-year risk factors for inferior knee injury and osteoarthritis outcome score after anterior cruciate ligament reconstruction: a study of 874 patients from the swedish national knee ligament register. Am J Sports Med.

[18] Hanypsiak BT, Spindler KP, Rothrock CR, Calabrese GJ, Richmond B, Herrenbruck TM (2008). Twelve-year follow-up on anterior cruciate ligament reconstruction:long-term outcomes of prospectively studied osseous and articular injuries. Am J Sports Med.

[19] Hjermundrud V, Bjune TK, Risberg MA, Engebretsen L, Aroen A (2010). Full-thickness cartilage lesion do not affect knee function in patients with ACL injury. Knee Surg Sports Traumatol Arthrosc.

[20] Imade S, Kumahashi N, Kuwata S, Kadowaki M, Tanaka T, Takuwa H (2013). A comparison of patient-reported outcomes and arthroscopic findings between drilling and autologous osteochondral grafting for the treatment of articular cartilage defects combined with anterior cruciate ligament injury. Knee.

[21] Lebel B, Hulet C, Galaud B, Burdin G, Locker B, Vielpeau C (2008). Arthroscopic reconstruction of the anterior cruciate ligament using bone-patellar tendon-bone autograft: a minimum 10-year follow-up. Am J Sports Med.

[22] Magnussen RA, Verlage M, Flanigan DC, Kaeding CC, Spindler KP (2015). Patient-reported outcomes and their predictors at minimum 10 years after anterior cruciate ligament reconstruction: a systematic review of prospectively collected data. Orthop J Sports Med.

[23] Murray JRD, Lindh AM, Hogan NA, Trezies AJ, Hutchinson JW, Parish E (2011). Does anterior cruciate ligament reconstruction lead to degenerative disease? Thirteen-year results after bone-patellar tendon–bone autograft. Am J Sports Med.

[24] Oiestad BE, Holm I, Aune AK, Gunderson R, Myklebust G, Engebretsen L (2010). Knee function and prevalence of knee osteoarthritis after anterior cruciate ligament reconstruction: a prospective study with 10 to 15 years of follow-up. Am J Sports Med.

[25] Oiestad BE, Holm I, Engebretsen L, Risberg MA (2011). The association between radiographic knee osteoarthritis and knee symptoms, function and quality of life 10–15 years after anterior cruciate ligament reconstruction. Br J Sports Med.

[26] Pike AN, Patzkowski JC, Bottoni CR (2019). Meniscal and chondral pathology associated with anterior cruciate ligament injuries. J Am Acad Orthop Surg.

[27] Risberg MA, Oiestad BE, Gunderson R, Aune AK, Engebretsen L, Culvenor A (2016). Changes in knee osteoarthritis, symptoms, and function after anterior cruciate ligament reconstruction: a 20-year prospective follow-up study. Am J Sports Med.

[28] Roos EM, Lohmander LS (2003). The knee injury and osteoarthritis outcome score (KOOS): from joint injury to osteoarthritis. Health Qual Life Outcomes.

[29] Rotterud JH, Risberg MA, Engebretsen L, Aroen A (2012). Patients with focal full-thickness cartilage lesions benefit less from ACL reconstruction at 2–5 years follow-up. Knee Surg Sports Traumatol Arthrosc.

[30] Rotterud JH, Sivertsen EA, Forssblad M, Engebretsen L, Aroen A (2016). Effect on patient-reported outcomes of debridement or microfracture of concomitant full-thickness cartilage lesions in anterior cruciate ligament-reconstructed knees: a nationwide cohort study from norway and sweden of 357 patients with 2-year follow-up. Am J Sports Med.

[31] Saris D, Price A, Widuchowski W, Bertrand-Marchand M, Caron J, Drogset JO (2014). Matrix-applied characterized autologous cultured chondrocytes versus microfracture: two-year follow-up of a prospective randomized trial. Am J Sports Med.

[32] Saris DB, Vanlauwe J, Victor J, Almqvist KF, Verdonk R, Bellemans J (2009). Treatment of symptomatic cartilage defects of the knee: characterized chondrocyte implantation results in better clinical outcome at 36 months in a randomized trial compared to microfracture. Am J Sports Med.

[33] Saris DB, Vanlauwe J, Victor J, Haspl M, Bohnsack M, Fortems Y (2008). Characterized chondrocyte implantation results in better structural repair when treating symptomatic cartilage defects of the knee in a randomized controlled trial versus microfracture. Am J Sports Med.

[34] Shelbourne KD, Gray T (2009). Minimum 10-year results after anterior cruciate ligament reconstruction: how the loss of normal knee motion compounds other factors related to the development of osteoarthritis after surgery. Am J Sports Med.

[35] Spindler KP, Huston LJ, Chagin KM, Kattan MW, Reinke EK, Amendola A (2018). Ten-year outcomes and risk factors after anterior cruciate ligament reconstruction: a MOON longitudinal prospective cohort study. Am J Sports Med.

[36] Tahami SM, Rad SM (2015). Outcome of ACL reconstruction and concomitant articular injury treatment. Arch Bone Joint Surg.

[37] Thrush C, Porter TJ, Devitt BM (2018). No evidence for the most appropriate postoperative rehabilitation protocol following anterior cruciate ligament reconstruction with concomitant articular cartilage lesions: a systematic review. Knee Surg Sports Traumatol Arthrosc.

[38] Ulstein S, Aroen A, Engebretsen L, Forssblad M, Lygre SHL, Rotterud JH (2018). Effect of concomitant cartilage lesions on patient-reported outcomes after anterior cruciate ligament reconstruction: a nationwide cohort study from Norway and Sweden of 8470 patients with 5-year follow-up. Orthop J Sports Med.

[39] Ulstein S, Årøen A, Engebretsen L, Forssblad M, Lygre SHL, Røtterud JH (2018). A Controlled comparison of microfracture, debridement, and no treatment of concomitant full-thickness cartilage lesions in anterior cruciate ligament-reconstructed knees: a nationwide prospective cohort study from Norway and Sweden of 368 patients with 5-year follow-up. Orthop J Sports Med.

[40] Ulstein S, Bredland K, Aroen A, Engebretsen L, Rotterud JH (2016). No negative effect on patient-reported outcome of concomitant cartilage lesions 5–9 years after ACL reconstruction. Knee Surg Sports Traumatol Arthrosc.

[41] Van Assche D, Staes F, Van Caspel D, Vanlauwe J, Bellemans J, Saris DB (2010). Autologous chondrocyte implantation versus microfracture for knee cartilage injury: a prospective randomized trial, with 2-year follow-up. Knee Surg Sports Traumatol Arthrosc.

[42] Van Assche D, Van Caspel D, Vanlauwe J, Bellemans J, Saris DB, Luyten FP (2009). Physical activity levels after characterized chondrocyte implantation versus microfracture in the knee and the relationship to objective functional outcome with 2-year follow-up. Am J Sports Med.

[43] Vanlauwe J, Saris DB, Victor J, Almqvist KF, Bellemans J, Luyten FP (2011). Five-year outcome of characterized chondrocyte implantation versus microfracture for symptomatic cartilage defects of the knee: early treatment matters. Am J Sports Med.

[44] Visna P, Pasa L, Cizmár I, Hart R, Hoch J (2004). Treatment of deep cartilage defects of the knee using autologous chondrograft transplantation and by abrasive techniques–a randomized controlled study. Acta Chir Belg.

[45] Weekes D, Campbell RE, Shi WJ, Ciccotti M, Salvo J, Cohen S (2020). Are patient and surgeon expectations after ACL reconstruction realistic?. Clin Orthop Relat Res.

[46] Widuchowski W, Widuchowski J, Koczy B, Szyluk K (2009). Untreated asymptomatic deep cartilage lesions associated with anterior cruciate ligament injury. Am J Sports Med.

[47] Zamborsky R, Danisovic L (2020). Surgical techniques for knee cartilage repair: an updated large-scale systematic review and network meta-analysis of randomized controlled trials. Arthroscopy.

